# Roles of Major RNA Adenosine Modifications in Head and Neck Squamous Cell Carcinoma

**DOI:** 10.3389/fphar.2021.779779

**Published:** 2021-11-25

**Authors:** Xing-xing Huo, Shu-jie Wang, Hang Song, Ming-de Li, Hua Yu, Meng Wang, Hong-xiao Gong, Xiao-ting Qiu, Yong-fu Zhu, Jian-ye Zhang

**Affiliations:** ^1^ Experimental Center of Clinical Research, Scientific Research Department, The First Affiliated Hospital of Anhui University of Chinese Medicine, Hefei, China; ^2^ Anhui Province Key Laboratory of Medical Physics and Technology, Institute of Health and Medical Technology, Hefei Institutes of Physical Science, Chinese Academy of Sciences, Hefei, China; ^3^ Department of Biochemistry and Molecular Biology, School of Integrated Chinese and Western Medicine, Anhui University of Chinese Medicine, Hefei, China; ^4^ Institute of Chinese Medical Sciences, State Key Laboratory of Quality Research in Chinese Medicine, University of Macau, Macao, China; ^5^ Key Laboratory of Molecular Target and Clinical Pharmacology and the State Key Laboratory of Respiratory Disease, School of Pharmaceutical Sciences and the Fifth Affiliated Hospital, Guangzhou Medical University, Guangzhou, China

**Keywords:** RNA modification, N 6-methyladenosine, N 1-methyladenosine, alternative polyadenylation, adenosine-to-inosine editing, head and neck squamous cell carcinoma, immunotherapy

## Abstract

Head and neck squamous cell carcinoma (HNSCC) is the sixth most common cancer malignancy worldwide and is known to have poor prognosis. The pathogenesis behind the development of HNSCC is not fully understood. Modifications on RNA are involved in many pathophysiological processes, such as tumor development and inflammation. Adenosine-related RNA modifications have shown to be linked to cancer and may play a role in cancer occurrence and development. To date, there are at least 170 different chemical RNA modifications that modify coding and non-coding RNAs (ncRNAs). These modifications affect RNA stability and transcription efficiency. In this review, we focus on the current understanding of the four major RNA adenosine modifications (N^6^-Methyladenosine, N^1^-Methyladenosine, Alternative Polyadenylation Modification and A-to-I RNA editing) and their potential molecular mechanisms related to HNSCC development and progression. We also touch on how these RNA modifications affect treatment of HNSCCs.

## Introduction

Head and neck squamous cell carcinoma (HNSCCs) mainly occurs in the mucosal epithelium of the oral cavity, pharynx or larynx. It is the sixth most common cancer worldwide, with over 800,000 cases diagnosed annually and an increasing incidence (Ferlay et al., 2019; Sung et al., 2021). Exposure to tobacco-derived carcinogens, excessive alcohol consumption, human papillomavirus and EBV are some of the triggers associated with HNSCC (Johnson et al., 2020). The molecular mechanisms behind HNSCC pathogenesis and development have not been fully elucidated. In recent years, advances in molecular biology expanded the understanding of epigenetics, especially post-transcriptional modifications of RNA, which play important roles in cell fate determination, proliferation, metabolism and many pathological processes.

**FIGURE 1:**
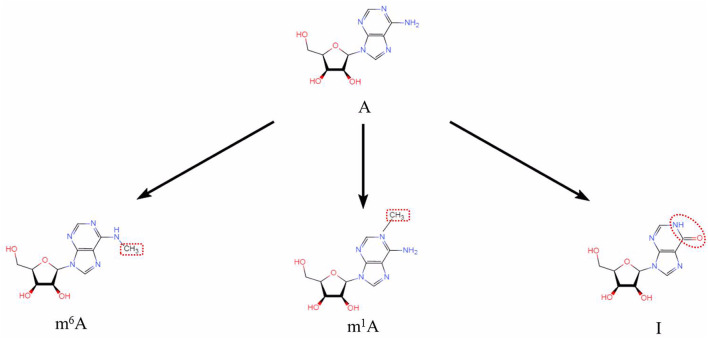
Three adenine-associated RNA modifications (N^6^-methyladenosine (m^6^A), N^1^-methyladenosine (m^1^A) and Adenosine to Inosine (A to I)). Chemical structures of three adenine-associated RNA modifications are shown.

**FIGURE 2:**
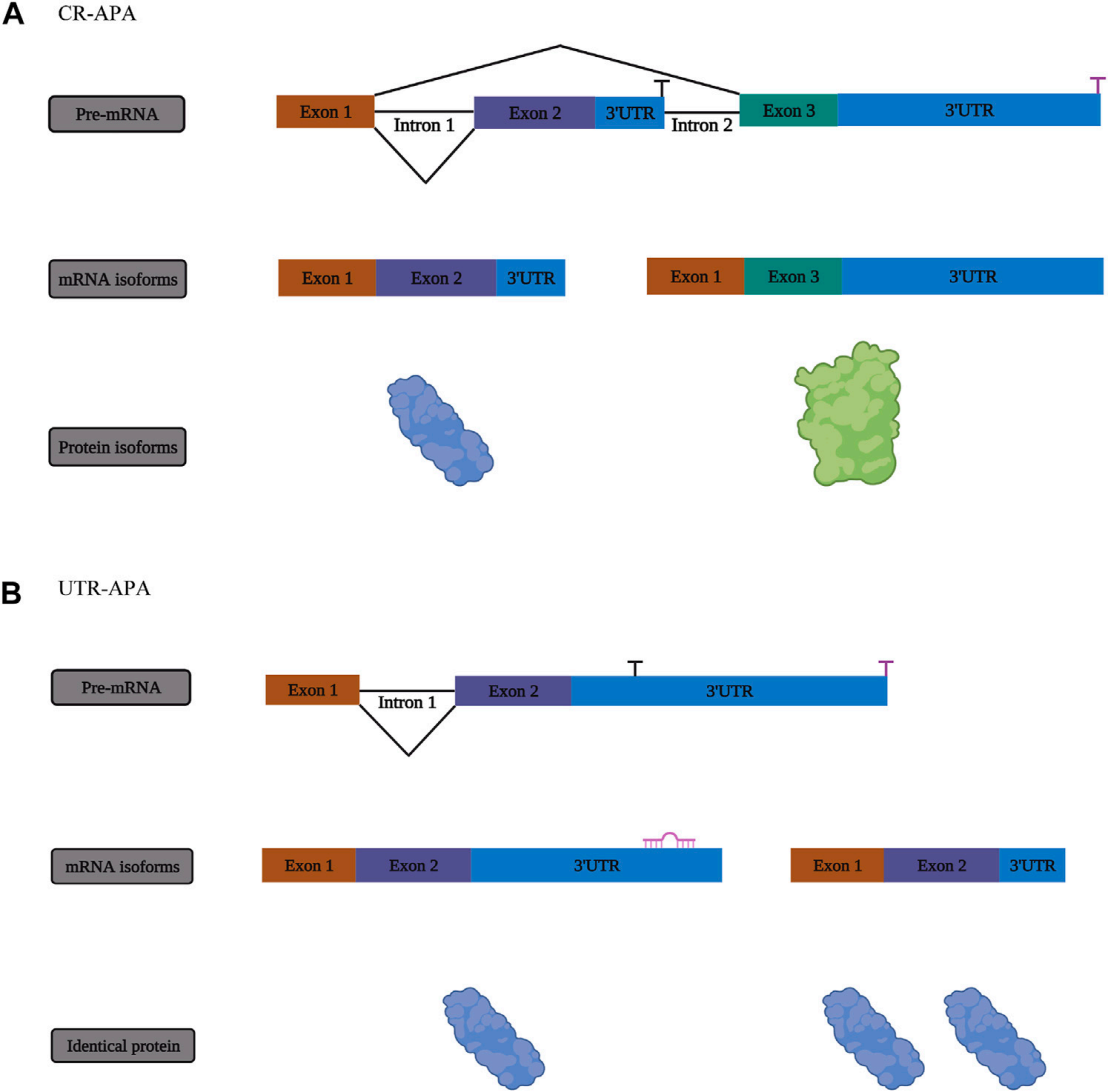
The mechanism of alternative polyadenylation (APA). **(A)** One form of APA, termed coding region alternative polyadenylation (CR-APA), occurs when the alternative polyA sites (PASs) are located within exons or introns. **(B)** Another form of APA is known as untranslated region alternative polyadenylation (UTR-APA) when alternative PASs are located in different regions of the 3′-UTR, resulting in the identical protein isoforms.

New messenger RNA (mRNA) transcripts require additional processing and modifications before translation and protein synthesis. RNA modifications regulate most steps of gene expression, from indirectly controlling DNA transcription through transcription factors, to directly affecting mRNA translation (Delaunay and Frye, 2019). Many RNA modifications have been uncovered thanks to next generation sequencing technologies. Some RNA modifications are difficult to study since there may be an inability to distinguish between certain nucleotides (Li et al., 2019c). To date, there are at least 170 different chemical RNA modifications known to modify coding and non-coding RNAs (ncRNAs) (Ramanathan et al., 2016; Xu et al., 2017; Dimitrova et al., 2019). The heavy nucleotide adenine is the most commonly modified in RNA. In this review, we focus on adenine-associated RNA modifications, including m^6^A methylation, m^1^A methylation, A-to-I RNA editing (Figure 1) and APA (Figure 2). We also discuss adenine-associated RNA modifications in regulating gene expression in HNSCC.

## Adenine-Related RNA Modifications

### N^6^-Methyladenosine Modifications

**FIGURE 3:**
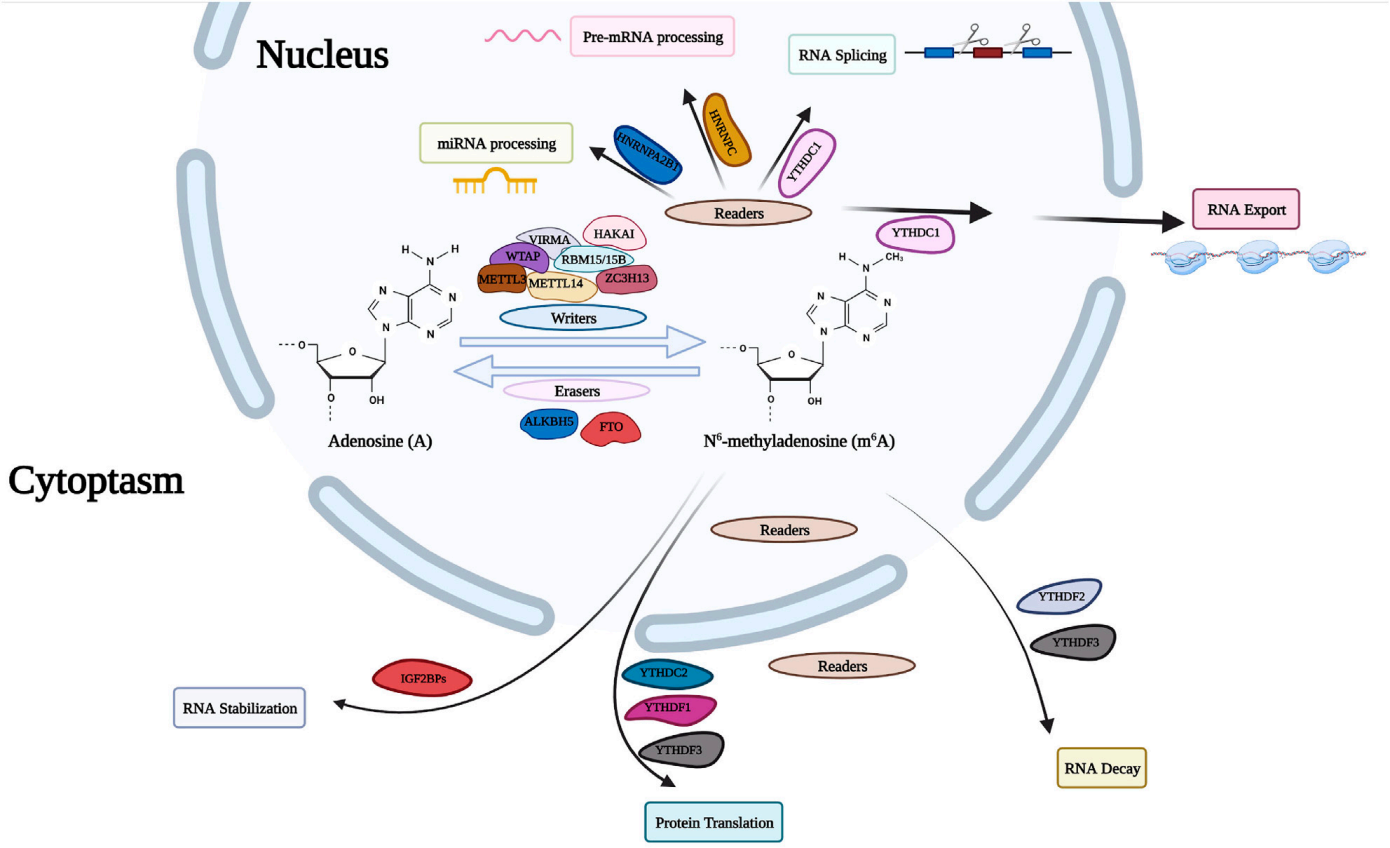
m^6^A RNA modifying process. The RNA modification of m^6^A is performed by the writer proteins with a polymer complex containing WTAP, HAKAI, METTL3, METTL14, ZC3H13, RBM15, and VIRMA, while the modification can be removed by the eraser proteins (FTO and ALKBH5). The modification is identified by reader proteins including YT521B homology (YTH) domain-containing protein family, HNRNP family and IGF2BPs. WTAP, Wilms Tumor 1 Associated Protein; HAKAI, Cbl Proto-Oncogene Like 1; METTL3, methyltransferase Like 3; METTL14, methyltransferase Like 14; ZC3H13, Zinc Finger CCCH-Type Containing 13; RBM15, RNA Binding Motif Protein 15; VIRMA, Vir Like m^6^A methyltransferase Associated; FTO, Fat Mass And Obesity-Associated Protein; ALKBH5, AlkB Homolog 5; HNRNP, Heterogeneous Nuclear Ribonucleoprotein; IGF2BP, Insulin Like Growth Factor 2 MRNA Binding Protein.

m^6^A is the most abundant and well-defined internal modification in mRNA. Methylation of the sixth nitrogen atom of RNA base A affects RNA stability and translation efficiency (Figure 3). This modification is programmed by m^6^A-methyltransferases, including methyltransferase-like 3 (METTL3), methyltransferase-like 14 (METTL14), WTAP, RNA-binding motif protein 15 (RBM15), RNA-binding motif protein 15B (RBM15B), ZC3H13 and KIAA1429.

### N6-Methyladenosine Writers

The m^6^A modification process undergoes first-order catalysis involving the two methyltransferase complexes (writers) METTL14 and METTL3. METTL3 coverts adenosine to m^6^A through its methyltransferase domain, and METTL14 is responsible for the recognition of RNA substrates. ZC3H13, RBM15 and VIRMA are also incorporated into the methyltransferase complex to regulate METTL14 and METTL3 functions.

### N6-Methyladenosine Erasers

The deposition of N^6^-methyladenosine on RNA is reversible through coordination of methyltransferases and demethylases. Fat mass and obesity-associated protein (FTO) and AlkB homolog 5 (ALKBH5) have been identified as members of non-heme Fe (II)/α -ketoglutarate-dependent dioxygenases. AlkB homolog 3 (ALKBH3), another member of the family, preferentially acts on m^6^A in tRNAs. The mechanisms where m^6^A methylation selectively and dynamically targets specific regions of the transcriptome are not fully understood. It is anticipated that additional m^6^A demethylases will be discovered.

### N6-Methyladenosine Readers

m^6^A modifications are unique recognition elements that bind proteins to readers and drive biochemical processes that occur in labeled RNA. The RNA-binding domain or the YTH domain contains YTH-domain proteins 1 and 2 family members (YTHDF1, YTHDF2, YTHDF3, YTHDC1 and YTHDC2). More recently, other readers have been identified, including insulin-like growth factor 2 mRNA-binding proteins (IGF2BP1, IGF2BP2, and IGF2BP3) and heterogeneous ribonucleic proteins (HNRNPC and HNRNPA2/B1). These proteins are highly expressed in different cancers and are involved in various molecular mechanisms. These proteins are not always dependent on the recognition of m^6^A.

### N^1^-Methyladenosine Modifications

m^1^A is a reversible modification produced when a methyl group attaches to the N^1^ position of adenosine. TRMT61A, TRMT61B, TRMT10C and TRMT6 have the ability to generate m^1^A modifications. It carries a positive charge when in physiological conditions and blocks the Watson–Crick interface, alters the structure of RNA, regulates protein-RNA interactions and affects the tertiary structure of ribosomes and translation (Shi et al., 2019). In mammals, the abundance of m^1^A in tRNAs and rRNAs is significantly greater than the abundance of mRNAs. Most m^1^A sites are located in the 5′-untranslated region (5′-UTR) of RNA (Dominissini et al., 2016). This modification affects both the occurrence and development of tumors by regulating gene expression and related biological processes.

### N^1^-Methyladenosine Writers

m^1^A “writers” contain a methyltransferase complex, including TRMT6, TRMT61A, TRMT10C and TRMT61B (Safra et al., 2017; Dai et al., 2018). In eukaryotes, m^1^A methyltransferases consist of TRMT6 and TRMT61A responsible for m^1^A58 modifications of cytoplasmic tRNAs (Ozanick et al., 2005). TRMT6 plays a key role in tRNA binding (Anderson et al., 2000). Studies have shown that TRMT10C and TRMT61B catalytic sites are located at positions 9 and 58 of human mt-tRNAs, respectively (Chujo and Suzuki, 2012; Vilardo et al., 2012). These positions on mt-tRNAs also coincide with the presence of m^1^A modifications (Ozanick et al., 2005). In addition, the tRNA m^1^A enzyme also modifies m^1^A modifications in mRNAs. For example, TRMT6/61A programs m^1^A sites in some nuclear mRNAs with GUUCRA tRNA-like motifs, while TRMT61B methylates half of the known m^1^A sites in mt-mRNAs (Li et al., 2017). Safra et al. also revealed that position 1374 of the mt-mRNA ND5 can be programmed into m^1^A by TRMT10C (Safra et al., 2017). Additional m^1^A mRNA enzymes may be discovered in the future.

### N^1^-Methyladenosine Erasers

m^1^A “erasers,” such as m^1^A demethylase, remove methyl groups from m^1^A. ALKBH1 and ALKBH3 both exert m^1^A demethylase activity and act as “erasers”. Li et al. proposed that ALKBH1 and ALKBH3, both members of the ALKB family, participate in demethylation of m^1^A sites in RNA (Li et al., 2016). Knocking out ALKBH1 increases m^1^A methylation (Liu et al., 2016). Knockout of ALKBH3 increases m^1^A levels in tRNAs and reduces protein synthesis in cancer cells (Ueda et al., 2017).

### N^1^-Methyladenosine Readers

The known “readers” for m^1^A methylation include YTHDF1, YTHDF2, YTHDF3 and YTHDC1, which decode m^1^A methylation markers and regulate m^1^A-related functions by mediating post-transcriptional regulation (Dai et al., 2018). A recent study revealed YTHDF1-3 and YTHDC1 as m^1^A readers, but not YTHDC2 (Dai et al., 2018). Electrophoretic mobility studies indicated that the YTH domain of YTHDF1-3 and YTHDC1 binds to RNA with m^1^A modifications. In contrast, the YTH domain of YTHDC2 does not appear to bind to RNA with m^1^A modification. Proteins containing an YTH domain recognize m^1^A modifications, proving that YTHDF1-3 and YTHDC1 may be readers of m^1^A modifications. However, there is limited research on m^1^A “readers”, and more m^1^A “readers” are expected to be uncovered in the future.

### Alternative Polyadenylation Modifications

Transcripts containing 3′-untranslation (3’-UTR) or coding regions of different lengths are generated through alternative polyadenylation (APA) when poly (A) tails are added or removed to different sites on RNA (Sandberg et al., 2008; Tian and Manley, 2017; Ikeda et al., 2020) (Figures 2A,B). Different transcripts have variable 3′-UTR lengths as a result of polyA sites, which contributes to polymorphisms (Pan et al., 2008; Wang et al., 2008; Xia et al., 2014). During embryonic development, APA is present at large amounts on mRNA. The most common form present in differentiated cells is the long 3′-UTR version (Grassi et al., 2018).

APA can affect the transcription in multiple ways (Lin et al., 2012). First, APA changes the position of protein products, adjusting metabolism (Berkovits and Mayr, 2015). Second, some APAs are tissue-specific and quickly respond to signals to regulate gene expression (Passacantilli et al., 2017). Furthermore, APA plays an important role in post-transcriptional splicing and can produce abnormal protein isoforms (Meyer et al., 2017; Ma et al., 2018; Zhu et al., 2018). In addition, upstream mechanisms, such as RNA processing factors and binding proteins, regulate APA and ultimately downstream biological processes (Zheng and Tian, 2014).

The proximal poly (A) site is used to form mRNAs with short 3 UTRs in highly proliferating cells (Ji and Tian, 2009). Studies have shown that NUDT21 plays an important role in bladder cancer. NUDT21 regulates the expression of ANXA2 and LIMK2 through Wnt/beta-catenin and NF-kappaB signaling pathways. ANXA2 and LIMK2 act by APA (Wang et al., 2019a). Among the various risk factors related to the occurrence and development of cancer, APA is an important endogenous factor directly triggering malignant phenotypes. Specific APA events are closely related to the occurrence of malignant tumors and autoimmune diseases (Rajasekar et al., 2020). For example, high levels APA in cancer cells are often accompanied by loss of 3′-UTR inhibitory elements, indicating that APA plays a universal role in oncogene activation (Gruber and Zavolan, 2019). By studying the regulatory factors or mediators during the APA process, new diagnostic criteria or therapeutic targets for cancer and other diseases may be identified.

### Adenosine-to-Inosine Editing

Epigenetic and post transcriptional mechanisms play important roles in gene expression and normal physiology editing is a post-transcriptional mechanism that changes the sequence of transcriptional RNA through post transcriptional modifications (Simpson and Emeson, 1996).

The most common form of RNA editing in higher eukaryotes includes A-to-I RNA editing, which occurs in double stranded RNA (dsRNA) where adenosine (A) forms inosine (I) through hydrolytic deamination (Roth et al., 2019). A-to-I editing alters amino acid sequences and affects other transcriptional processes, thereby promoting tumorigenesis and tumor progression through site-specific modifications of tumor-associated genes (Chen et al., 2017b; Hong et al., 2018; Xu et al., 2019a; Han et al., 2020). Adenosine deaminase (ADARs), including ADAR1, ADAR2 and ADAR3, catalyze this reaction (Valente and Nishikura, 2005; Yao et al., 2019). The ADAR family all contain a highly conserved C-terminal catalytic deaminase domain and several N-terminal dsRNA binding domains (Zinshteyn and Nishikura, 2009). The mammalian ADAR family consists of three structurally conserved members, ADAR1, ADAR2 and ADAR3 (Bass, 2002). ADAR1 and ADAR2 are expressed in most tissues and their catalytic activity has been observed for a while (Lehmann and Bass, 2000; Wong et al., 2001). ADAR3 is highly expressed in the central nervous system (Chen et al., 2000; Wang et al., 2019b). The ADAR1 protein has both a long (P150) and short (P110) isoform. ADAR1p110 is predominantly found in the nucleus, whereas ADAR1p150 is found in both the nucleus and cytoplasm. ADAR1p150 expression is driven by an interferon inducible promoter and is upregulated in situations of cellular stress or viral infection (Patterson and Samuel, 1995). ADAR1 plays an important role in A-to-I RNA editing and influences cancer development (Qin et al., 2014; Heraud-Farlow et al., 2019; Vlachogiannis et al., 2021). ADAR2 is present in the nucleus of cells and ADAR2 along with ADAR1 may both influence the function of neurons (Behm et al., 2017; Hosaka et al., 2019). ADAR3 exists as a monomer *in vitro* and is specifically expressed only in brain tissue, which may explain why it is thought to be catalytically inactive (Chen et al., 2000; Nishikura, 2010).

## Adenine-Related RNA Modification Regulation in Head and Neck Squamous Cell Carcinoma

### The Role of N^6^-Methyladenosine in Head and Neck Squamous Cell Carcinoma

**FIGURE 4:**
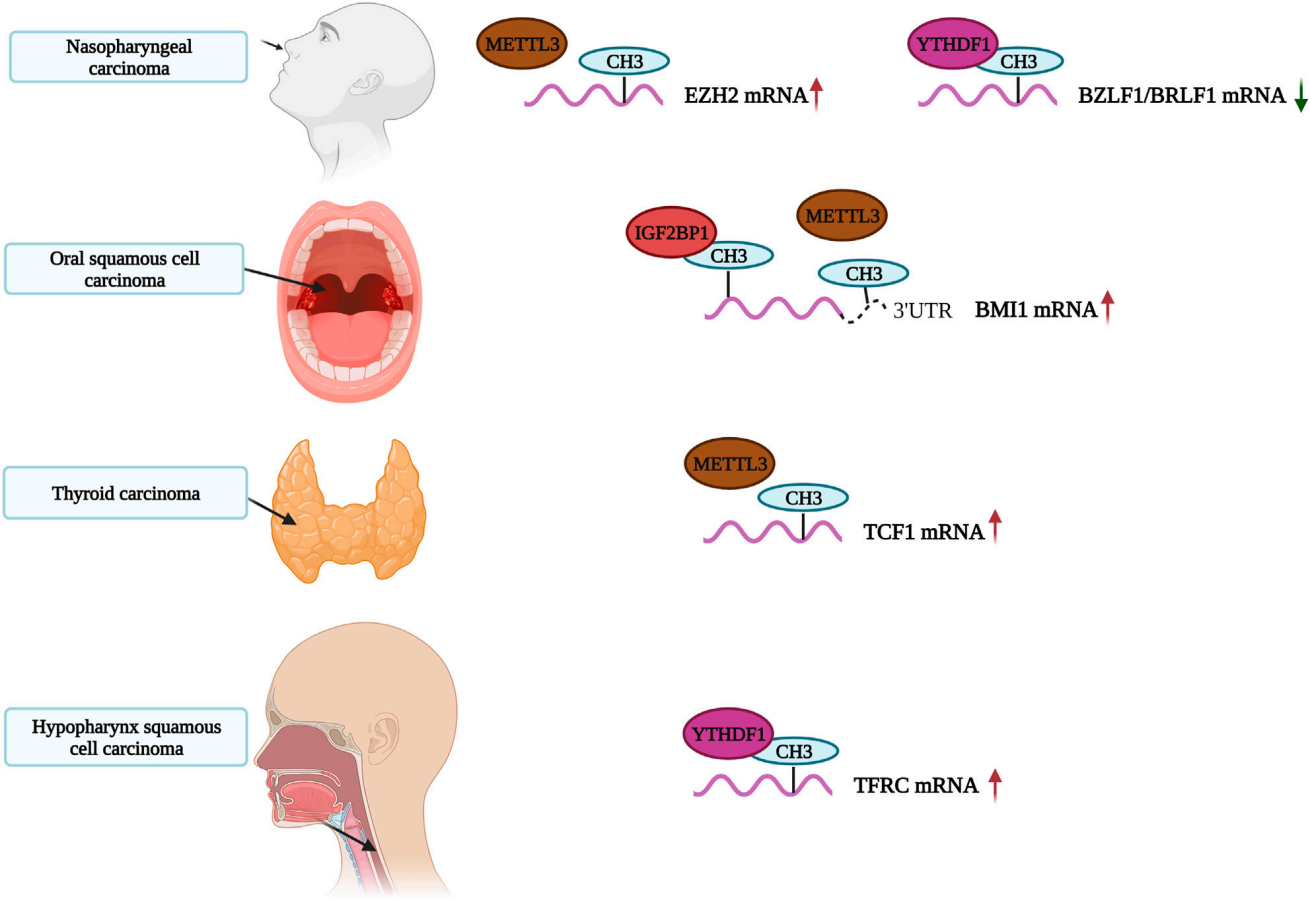
The role of m^6^A in Head and Neck Carcinoma. The m^6^A-modified RNA play an important role in the occurrence and development of Head and Neck Carcinoma.

Presently, it is generally believed that m^6^A methylation modifications play key roles in the occurrence and development of carcinomas. Most studies indicate that an increase of m^6^A methylation levels promotes tumor growth, development and treatment resistance (Figure 4). Here, we review recent articles studying m^6^A modifications in head and neck carcinoma. A summary of these compiled studies are presented in Table 1.

**TABLE 1:** Regulation of m^6^A modification in HNSCC.

m6A Regulators	Target	Regulation in HNSCC	Function	Mechanisms
METTL3 Wu et al. (2021); Arumugam et al. (2021); Ai et al. (2021)	Circux1 Wu et al. (2021)	Up	writer	METTL3 promotes the m6A methylation level of Circux1 and increases the stability of its expression in hypopharyngeal squamous cell carcinoma (HPSCC) Wu et al. (2021)
	PRMT5 and PD-L1 Ai et al. (2021)	Up	writer	METTL3 can promote the progress of OSCC by increasing the m6A editing degree of PRMT5 and PD-L1 Ai et al., 2021
METTL3 and METTL14	LNCAROD	Up	writer	METTL3 and METTL14 can stabilize the expression of LNCAROD in HNSCC cells through m6A modification Ban et al., 2020
RBM15	TMBIM6	Up	writer	RBM15 mediates the m6A modification of TMBIM6 and stabilizes the expression of TMBIM6 through IGF2BP3 Wang et al. (2021b)
KIAA1429 (VIRMA)	KIAA1429	Up	writer	KIAA1429 (VIRMA) can be used as a “writer” of m6A to help overexpression of KIAA1429 mRNA Arumugam et al., 2021
IGF2BP family (IGF2BP1, IGF2BP2 Geng et al., 2021; Paramasivam et al., 2021, IGF2BP3)	HMGA2	Up	reader	The IGF2BP family promotes tumor progression by reading m6A-modified oncogenic mRNA Paramasivam et al., 2021
	TK1			
	HDGF			
	FSCN1			
	MKI67			
	CD44			
YTHDF1 Ye et al., 2020; Zhou et al., 2020		Up	reader	The YTHDF1 methyltransferase domain can bind to the 3′UTR and 5′UTR of TRFC mRNA to promote m6A modification and translation of TFRC mRNA Ye et al., 2020
YTHDF2 Zhou et al., 2020				
YTHDF3 Arumugam et al., 2021				
YTHDC2 Zhou et al., 2020			reader	
ALKBH5 Shriwas et al., 2020	FOXM1, NANOG (Shriwas et al., 2020)	Up	eraser	DDX3 directly regulates ALKBH5 to eliminate m6A methylation in the new transcripts of FOXM1 and NANOG Shriwas et al., 2020
FTO Zhou et al., 2020; Paramasivam et al., 2021			eraser	
	GRHL3-AS1, AL121845.4, AC116914.2, AL513190.1	Down		GRHL3-AS1, AL121845.4, AC116914.2, AL513190.1 have a protective effect on HNSCC patients

### N6-Methyladenosine Writers in Head and Neck Squamous Cell Carcinoma

METTL3 is a common writer for m^6^A methylation. It is highly expressed in gastric, bladder, colorectal and pancreatic cancers as well as, glioblastoma and other tumors. High levels of METTL3 indicate a poor prognosis for gastric cancer (Li et al., 2019a; Li et al., 2019b; Han et al., 2019; Xia et al., 2019; Yue et al., 2019; Zhao and Cui, 2019; Wang et al., 2020a). METTL3 also plays an important role in head and neck carcinoma. A study by Wu et al. found that METTL3 regulates the m^6^A methylation levels of circCUX1 and increases its stability in hypopharyngeal squamous cell carcinoma patients resistant to radiotherapy. The circCUX1 further inhibits caspase1 expression, reduces the release of inflammatory factors and increases the tolerability of tumor cells to radiotherapy. Knockout of circCUX1 increases the sensitivity of hypopharyngeal cancer cells to radiotherapy (Wu et al., 2021). Similarly, Ai et al. confirmed that METTL3 is highly expressed in oral squamous cell carcinoma (OSCC). METTL3 writes m^6^A modifications in PRMT5 and PD-L1 and promotes the progression of OSCC by increasing m^6^A editing (Ai et al., 2021). Interestingly, Liu et al. also found that the expression of METTL3 in two OSCC cohorts was significantly higher than in normal adjacent tissues, which was associated with a poor prognosis. METTL3 promotes proliferation, migration and invasion of OSCC *cells in vitro* by mediating m^6^A modifications in the 3′-UTR of BMI1 mRNAs. METTL3 also cooperates with IGF2BP1 to promote the translation of BMI1 in OSCC. The METTL3-m^6^A-BMI1 axis may serve as a therapeutic target or prognostic biomarker for OSCC (Liu et al., 2020). Meng et al. found that METTL3 was highly expressed in nasopharyngeal carcinoma (NPC) tissues, with increasing levels of expression associated with tumor stage. METTL3 is associated with the vitality and migration ability of NPC cells. In terms of mechanism, METTL3 binds and mediates m^6^A modifications on EZH2 mRNAs, inhibiting EZH2 expression and upregulating CDKN1C expression. This increases the malignancy and promotes the development of NPC (Meng et al., 2020). We speculate that the METTL3-EZH2-CDKN1C regulatory axis may be closely related to NPC, and m^6^A methylation modifications affect its activation.

METTL3 and METTL14 also mediate m^6^A modifications. Ban et al. found that METTL3 and METTL14 stabilize or even increase the expression of LNCAROD in HNSCC cells through m^6^A modifications. LNCAROD binds to YBX1 and HSPA1A proteins, and its overexpression enhances the proliferation and migration of HNSCC cells. The dysregulation of m^6^A methylation may cause abnormal expression of LNCAROD in HNSCC (Ban et al., 2020). In laryngeal squamous cell carcinoma (LSCC) patients, the expression of is significantly increased RBM15, indicating a poor prognosis. RBM15 mediates m^6^A modifications of TMBIM6, and stabilizes the expression of TMBIM6 through IGF2BP3, thereby promoting LSCC (Wang et al., 2021b). Arumugam et al. reported that the expression levels of KIAA1429 in HNSCC tissues were significantly higher than in normal tissues. The increase of KIAA1429 expression is closely related to HNSCC metastasis. KIAA1429 (VIRMA), an m^6^A writer, is frequently amplified and mutated (8%), which promotes the overexpression of KIAA1429 mRNA. Therefore, the methylation level of KIAA1429 (VIRMA) may be closely related to HNSCC (Arumugam et al., 2021). Similarly, there is evidence that KIAA1429 is a highly expressed m^6^A regulatory gene in HNSCC (Zhao and Cui, 2019; Paramasivam et al., 2021). Unfortunately, these studies did not explore these relevant mechanisms in depth.

### N6-Methyladenosine Readers in Head and Neck Squamous Cell Carcinoma

The IGF2BP family promotes tumor progression by reading m^6^A-modified oncogenic mRNA. IGF2BP2 plays a key role in the progression of many cancers. Studies have shown that the overexpression of IGF2BP2 predicts poor prognosis of patients with colorectal cancer, acute myeloid leukemia and metaplastic breast cancer (He et al., 2018; Wang et al., 2020a). Upregulation of IGF2BP2 in pancreatic cancer may influence cell proliferation through the PI3K/Akt signaling pathway (Xu et al., 2019b). It has been confirmed that SNPs in IGF2BP2 and IGF2BP3 promote lymph node metastasis in esophagogastric junction adenocarcinoma (Chen et al., 2018). IGF2BP2 has also been linked to the occurrence and development of head and neck carcinoma. Paramasivam et al. pointed out that nearly half of HNSCC patients show significant changes in m^6^A regulatory genes (Paramasivam et al., 2021). Among the aforementioned patients, nearly half of the patients showed increased expression of IGF2BP2, and the expression of the most common oncogene in HNSCC patients increased with higher levels of IGF2BP2. Similarly, Geng et al. reported that IGF2BP2 is increased in HNSCC and serves as an m^6^A regulatory gene (Geng et al., 2021). This shows that IGF2BP2 is closely related to the occurrence and development of HNSCC. Deng et al. showed that IGF2BP2 is upregulated in HNSCC tissues, and its high expression is associated with poor prognosis, playing a key role in HNSCC progression (Deng et al., 2020). Wang et al. also found that IGF2BP2 is highly expressed in papillary thyroid cancer (PTC) tissues and is closely related to the poor prognosis of PTC patients. The risk score of the m^6^A-related IGF2BP2 signature can be used as an independent prognostic factor of PTC, which helps predict the disease-free survival of PTC patients (Wang et al., 2020d). This evidence indicates that the IGF2BP family may act as a key regulatory node, positively regulating the pathogenesis of HNSCC through m^6^A methylation modifications.

A recent study showed that the m^6^A reader YTH N6-methyladenosine RNA-binding protein 1 (YTHDF1) promotes the degradation of BZLF1 and BRLF1 by recruiting the RNA degrading proteins ZAP, DDX17 and DCP2. YTHDF1 ultimately inhibits EBV infection and lytic replication. This process is dependent on XRN1. Xia et al. speculated that EBV introduces m^6^A modifications into host cells to destabilize BZLF1 and BRLF1, thereby inhibiting lytic replication and maintaining the incubation period of the virus (Xia et al., 2021). Previously, Ye et al. demonstrated that high expression of YTHDF1 increases m^6^A modifications on TFRC mRNA to promote iron accumulation in hypopharyngeal squamous cell carcinoma (HPSCC). These modifications on TFRC mRNA have also influence tumor occurrence and proliferation. At the same time, YTHDF1 and iron-related genes (FTH1 and TFRC) are significantly upregulated in tumor tissues (Ye et al., 2020). Therefore, YTHDF1 may serve as a potential therapeutic target for HNSCC.

YTHDC2 is an m^6^A reader protein (Kretschmer et al., 2018). It is a tumor suppressor gene reduced in HNSCC tissues that is closely associated with prognosis and immune infiltration levels (Li et al., 2020a).

### N6-Methyladenosine Erasers in Head and Neck Squamous Cell Carcinoma

Only two m^6^A erasers, including FTO and ALKBH5, have been uncovered so far (Zhao and Cui, 2019; Zhou et al., 2020; Paramasivam et al., 2021). FTO, a m^6^A demethylase associated with human obesity, reverses m^6^A modifications (Jia et al., 2011). Shriwas et al. found that the m^6^A demethylase ALKBH5 is directly regulated by the DEAD-box RNA helicase 3 (DDX3), erasing m^6^A methylation in new FOXM1 and NANOG transcripts and causes chemotherapy resistance (Shriwas et al., 2020). FTO regulates the proliferation and migration of cervical cancer cells by modifying E2F1 and Myc transcripts (Zou et al., 2019). However, there is no related work investigating the role of FTO as an m^6^A eraser in HNSCC.

### Bioinformatics Reveal N6-Methyladenosine Methylation Regulatory Genes

Of the expression of many m^6^A regulatory genes, including writers (METTL3, METTL14, WTAP, ZC3H13 and RBM15), erasers (ALKBH5 and FTO), and readers (YTHDF1, YTHDF2, YTHDF3, YTHDC1, IGF2BP1 and IGF2BP3) were found to be significantly increased in HNSCC tissues (Feng et al., 2021; Paramasivam et al., 2021). Deng et al. pointed out that the expression of m^6^A methylation regulatory genes is significantly related to the prognosis of HNSCC (Deng et al., 2021). Zhou et al. identified 10 m^6^A regulators and those high expression levels of ALKBH5, FTO, METTL14, WTAP, YTHDC1, YTHDF1 and YTHDF2 predict poor prognosis for HNSCC patients. The expression level of YTHDC2 was also found to be directly proportional to patient prognosis (Zhou et al., 2020). However, this study also performed a preliminary analysis and further studies are needed. Feng et al. used bioinformatic methods to screen 4 m^6^A-modified differentially expressed lncRNAs between high-risk group and low-risk group cancer groups. Interestingly, results showed that the expression levels of these four m^6^A-modified lncRNAs were reduced in the high-risk group. The higher the m^6^A-modified lncRNA expression level, the higher the survival rate of HNSCC patients. In other words, these four m^6^A-modified lncRNAs showed protective effects for HNSCC patients (Feng et al., 2021). This finding differs from public opinion, but indicates that the biological role of m^6^A methylation modifications in HNSCC requires further exploration. Other relevant bioinformatic analyses show similar findings (Zhao and Cui, 2019; Li et al., 2020a; Huang et al., 2020). These studies identified key genes as potential aspects for future research and treatment (Yi et al., 2020).

### The Role of N^1^-Methyladenosine in Head and Neck Squamous Cell Carcinoma

There are limited studies investigating the role of m^1^A modifications in cancer as well its role in HNSCC. Some studies revealed that m^1^A methylation modifications influence different cancers including hepatocellular carcinoma (HCC), lung cancer, colorectal cancer and pancreatic cancer (Wang et al., 2020; Shi et al., 2020; Xie et al., 2020). In addition, Li et al. found that expression of m^1^A regulatory factors are significantly altered in cancer patients and are closely related to changes in carcinogenic pathways and overall survival rate (Li et al., 2021).

Zhao et al. comprehensively analyzed TCGA data for diagnosed with esophageal carcinoma (ESCA), liver hepatocellular carcinoma (LIHC), stomach adenocarcinoma (STAD), pancreatic adenocarcinoma (PAAD) and colorectal adenocarcinoma (COAD)). Expression of m^1^A regulators, such as writers (TRMT6, TRMT61A and TRMT10C), erasers (ALKBH1, ALKBH3) and readers (YTHDF1-3, YTHDC1), were significantly changed in all gastrointestinal cancers analyzed.

The expression levels of m^1^A regulatory genes are significantly higher in HCC than in normal tissues. In addition, the expression levels of TRMT6, TRMT61A and TRMT10C, as well as ALKBH3 and YTHDF2, are higher in patients with late-stage tumors (G1-G3) (Zhao et al., 2019). Shi et al. also found that the expression of m^1^A-related regulatory genes, such as TRMT6, TRMT61A, TRMT10C and YTHDF1, are helpful in assessing risk and survival prediction of HCC patients. A significant correlation between YTHDF1 and TRMT6 was identified in their study. YTHDF1, which acts as m^6^A modification reader, also acts as m^1^A modified RNA binding protein, playing an important role in regulating m^1^A methylation modifications. TRMT6 forms a methyltransferase complex with TRMT61A to catalyze the methylation of the N^1^ position of adenosine residues in mRNAs. Increased expression of TRMT6 is associated with poor prognosis (Shi et al., 2020). Meanwhile, Zhao et al. found that some GC patients show alterations in m^1^A regulatory factors, including mutations, copy number amplifications or deep deletions. The change frequency of TRMT6 is nearly 1.8%, and the mutation frequency of the YTHDF1 and YTHDF3 readers is highest amongst GC patients, reaching 6 and 5%, respectively (Zhao et al., 2019). This is consistent with findings by Shi et al. Their studies revealed that copy number variations (CNV) have a higher frequency in m^1^A-related regulatory genes. The differential expression of 10 m^1^A-related regulatory genes, such as the reader YTHDF1, can be used as prognostic indicators (Shi et al., 2020). Based on these observations, there is reason to believe that YTHDF1 can be used as a reader along with TRMT6 and TRMT61A to jointly regulate m^1^A methylation in HCC. Interestingly, Wang et al. also observed dysregulation in m^1^A regulatory factors in gynecological cancers. TRMT10C was found to be highly expressed in ovarian and cervical cancers and being associated with a poor prognosis. TRMT10C inhibits the proliferation and migration of ovarian and cervical cancer cells (Wang et al., 2020c). Therefore, it is reasonable to believe that TRMT10C can also be used as a biomarker for predicting the prognosis of patients with gynecological cancers. m^1^A modification-related genes may affect the expression of oncogenes through m^1^A methylation and may be involved in the progression of HNSCC.

These differences are also present in PAAD but only at a moderate level compared with HCC (Shi et al., 2020). Zheng et al. also found that changes of m^1^A regulatory genes in PAAD are associated with cancer stage. Some of these genes serve as writers or readers and have a high mutational frequency. CNV has also been found to have a high frequency of mutations. Meanwhile, changes in the YTHDF1 and TRMT61A genes were found in two PAAD samples. Even though high expression levels of YTHDF1 and YTHDF2 were found to be associated with the poor prognosis of PAAD patients (Chen et al., 2017a), it is not clear whether this is due tom^1^A regulation. The m^1^A-related writers, erasers and readers are differentially expressed depending on the stage. Low expression of the ALKBH1 eraser predicts poor prognosis of PAAD patients (Zheng et al., 2021). ALKBH1 contains both m^1^A and m^6^A demethylase activities (Wu et al., 2016; Kawarada et al., 2017). Wang et al. found that ALKBH1, YTHDF1, TRMT6, TRMT10C and TRMT61B are elevated in endometrial cancers (Wang et al., 2020c). Therefore, overexpression of ALKBH1 most likely reverses methylation and inhibits tumor development. Shi et al. identified that m^1^A modifications are increased in COAD tissues, accompanied by down-regulation in lncRNA expression levels relative to normal adjacent tissues (Shi et al., 2021). Meanwhile, three different m^1^A modification patterns identified by Gao et al. significantly affect relapse-free survival, overall survival and the number of tumor microenvironment infiltration cells in COAD patients. In addition, Pan et al. found that m^1^A regulatory factor expression levels significantly differed between lung squamous cell carcinoma and normal tissues (Pan et al., 2021).

This evidence proves that m^1^A methylation modifications play important roles in gastrointestinal, gynecological and lung cancers. m^1^A methylation modifications also influence the occurrence and development of HNSCC.

### The Role of Alternative Polyadenylation in Head and Neck Squamous Cell Carcinoma

APA induces 3′-UTR shortening and it has been considered a specific feature of tumorigenesis and involved in the development of HNSCC.

CPSF plays an important role in processing the 3′-processing complex. Core 3′-processing factors include protein complexes (CPSF, CSTF, CFI and CFII) and several single proteins. CPSF1 is the largest subunit of the protein complex (Murthy and Manley, 1995) that recognizes polyadenylation signals and regulates APA (Martin et al., 2012; MacDonald, 2019). Sakai et al. identified 13 candidate spliceosome genes significantly altered in HNSCC. In these candidate genes, a higher number of alternative splicing events (ASEs) were identified with CPSF1 was overexpressed. This promoted the growth of HNSCC cells. In addition, junction analysis showed that abnormal expression of CPSF1 is related to ASEs of cancer-related genes, such as LAMC2, UBE2C, AKT2, AKT2, BOK, MAP4 and FANCD2. Therefore, it was speculated that CPSF1 overexpression leads to abnormal splicing of oncogenes and promotes tumor occurrence and development (Sakai et al., 2020). Interestingly, there is evidence that CPSF1 promotes tumor development by regulating APA events in triple-negative breast cancer (TNBC). Wang et al. determined that CPSF1 and PABPN1 are the main C/P factors that regulate APA events. Knockout of CPSF1 or PABPN1 reverses APA events of tumor-related genes in TNBCs, inhibits tumor cell proliferation, promotes apoptosis and redistributes the cell cycle (Wang et al., 2020b). This indicates that the mechanism by which APA regulates TNBC cell proliferation may be achieved by changing core processing factor (CPSF1 and PABPN1) levels. We speculate that CPSF1 may affect tumors development and group through the APA pathway.

PABPN1 may also act as the core factor of APA regulation. Poly (A) tails grow to ∼250 nucleotides. PABPN1 binds to poly (A) and breaks the connection between CPSF and poly (A) polymerase, thereby controlling the length of the poly (A) tail (Wahle, 1995; Kuhn et al., 2009; Eckmann et al., 2011). Xiang et al. proved that PABPN1 is a key factor regulating the APA profile of many cancers (Xiang et al., 2018). Ichinose et al. found that PABPN1 acts as an APA inhibitor. Deletion of PABPN1 induces APA events, leading to microRNA-mediated gene regulation and causing the release of cancer cells (Ichinose et al., 2014). In summary, we speculate that CPSF1 and PABPN1 serve as key regulatory factors to increase APA events and promote HNSCC development.

In addition, we believe that some oncogenes alter the length of 3′-UTR through APA events, thereby evading gene suppression and promoting the growth of HNSCC (Martin et al., 2012; Missan et al., 2015). It has been demonstrated in NPC that the oncogene FND3CB increases proximal polyadenylation sites through APA events, resulting in shorter 3′-UTRs. This evades miRNA-mediated gene suppression (Li et al., 2020b). The same mechanism of action has also been identified in bladder cancer (Xiong et al., 2019) and HCC (Sun et al., 2017; Tan et al., 2018). Meanwhile, 195 genes were also identified in NPC and their tandem 3′-UTR lengths were significantly different between NPC and the control group (Xu et al., 2018). In esophageal squamous cell carcinomas, 903 genes related to adhesion junctions and the cell cycle shortened the 3′-UTR, and the distal PolyA site was used by 917 genes (Sun et al., 2014). This difference may be caused by APA-mediated gene expression regulation. In summary, APA may play a key role in the occurrence and development of HNSCC.

### The Role of Adenosine-to-Inosine Editing in Head and Neck Squamous Cell Carcinoma

Currently, there are only a few studies directly investigating A-to-I RNA editing in HNSCC. One reason for this may be due to limited detection technology in identifying A-to-I editing sites. Previous studies have shown A-to-I RNA editing (R334G) in the tumor suppressor gene prox1 present in 4 of 8 esophageal cancer cases, indicating that A-to-I RNA editing may be closely related to the progression of this cancer (Yoshimoto et al., 2007). Hochberg et al. identified two A-to-I editing sites in insulin-like growth factor-binding protein-7 (IGFBP7) transcripts. In the normal epidermis, IGFBP7 transcripts are highly edited, but this editing is significantly reduced in basal cell and squamous cell carcinomas. Edited IGFBP7 inhibits the proliferation of keratinocytes and induces their senescence. These results indicate that A-to-I RNA editing in IGFBP7 maintains the balance between normal skin proliferation and aging, and its reduction may promote the occurrence and development of carcinoma (Hochberg et al., 2013). A recent study showed that A-to-I SLC22A3 RNA editing resulted in a decrease in SLC22A3 gene expression and led to lymph node metastasis in ESCC cases. This process almost only occurs in familial high-risk individuals, making them susceptible to ESCC, but does not occur in sporadic ESCC cases. The A-to-I transcript editing event (A261 at exon 1) that resulted in the substitution of asparagine (Asn)-aspartate (Asp) amino acids may be related to ESCC susceptibility (Fu et al., 2017). However, this study later discovered that the RNA editing enzyme ADAR2 is a familial ESCC susceptibility gene, suggesting that ADAR plays a central role in A-to-I RNA editing. Adenosine deaminase catalysis that acts on the RNA (ADARs) family plays an important role in A-to-I RNA editing. Therefore, most current research is focused on ADARs.

### ADAR1 as a Foe

ADAR1 may be closely related to the occurrence and development of HNSCC. Zhang et al. showed that ADAR1 expression is related to STAT1, STAT2 and IRF9, and the abundance of ADAR1 protein is related to the activation of JAK/STAT pathway induced by type I interferons. The activation of JAK/STAT pathway regulates the expression of ADAR1, which leads to an abnormal RNA editing spectrum in ESCC (Zhang et al., 2017). Interestingly, Qin et al. found that ADAR1 was overexpressed in ESCC and that it predicted a poor prognosis. Over-editing of AZIN1 information catalyzed by ADAR1 makes carcinomas more aggressive. This study confirmed that ADAR1 can be used as an oncogene, and the excessive A-to-I editing mediated by it promotes the development of ESCC (Qin et al., 2014). In summary, ADAR1 may be closely related to the occurrence and development of ESCC. A-to-I RNA editing mediated by ADAR1 may promote ESCC. In addition, there is evidence that ADAR1 plays an important biological function in oral squamous cell carcinoma (OSCC). Liu et al. found that ADAR1 is over-expressed in OSCC and positively correlates with migration, invasion and EMT. ADAR1 may combine with dicer to increase the expression of oncogenic miRNAs, affect cell migration and invasion, promote carcinoma growth and reduce patient survival (Bo et al., 2019). Ma et al. found that ADAR binds to forkhead box D1 antisense RNA 1 (FOXD1-AS1) and FOXD1 in OSCC cells, to enhances the stability of FOXD1 mRNA, thereby promoting the occurrence and development of OSCC (Ma et al., 2021). This evidence indicates that ADAR1 is involved in A-to-I RNA editing to promote the occurrence and development of HNSCC. An increase in A-to-I RNA editing will lead to increased proliferation and migration, which indicates a poor prognosis for HNSCC patients.

### ADAR2: Friend or Foe?

Behm et al. found that mice do not survive to adulthood in the absence of the active editing enzymes ADAR1 or ADAR2. As neurons mature, the number of interactions between ADAR2 and nuclear isomerase Pin1 increases, which contributes to the stability of ADAR2 protein and helps mouse neurons develop and mature (Behm et al., 2017). Terajima et al. showed that ADAR2 mediated A-to-I RNA editing critically contributes to light induced circadian clock phase shifts in the suprachiasmatic nucleus of the mouse hypothalamus (Terajima et al., 2018). However, Agranat et al. identified arginine/glycine sites of SON mRNA as ADAR2 dependent sites. They also detected multiple circular RNAs with ADAR2 dependent sites in SH-SY5Y cells and culture medium. These RNAs with ADAR2 dependent sites may serve as biomarkers for amyotrophic lateral sclerosis (Agranat et al., 2010). As previously mentioned, Fu et al. determined that ADAR2 is a familial ESCC susceptibility gene and that SLC22A3 is a tumor suppressor gene. ADAR2 is overexpressed in familial normal esophageal tissues, which increases the A-to-I RNA editing of the SLC22A3 gene and reduces the expression of the SLC22A3 gene, thereby increasing the susceptibility of ESCC (Fu et al., 2017). Interestingly, Chen et al. believe that overexpression of ADAR2 inhibits the growth and induces apoptosis by editing and stabilizing IGFBP7 in ESCC. Knockout of ADAR2 in carcinoma cells with high expression of ADAR2 inhibits tumor cell apoptosis (Chen et al., 2017d). This is contrary to the view of Fu et al., which suggests that overexpression of ADAR2 promotes tumor growth (Fu et al., 2017). Therefore, additional studies are needed to identify the true roles of ADAR2.

### ADAR3: A Potential Friend

The biological role of ADAR3 has been studied over recent years. Wang et al. found that ADAR3 binds to two activity-dependent immediate-early genes in response to brain stimulation that encode the 3′-UTRs of the mRNAs early growth response 1 (EGR1) and bispecific phosphatase 1 (DUSP1), thereby regulating the transcription levels of DUSP1 and EGR1. This result suggests that ADAR3 may play a new role in brain function (Wang et al., 2019b). ADAR3 was also found to be related to Q/R locus editing. The Q/R site refers to the codon modification site of the glutamate receptor ionotropic AMPA 2 (GRIA2). GRIA2 is an adenosine in glutamate receptor subunit B transcripts. In glioblastoma, the reduction of GRIA2 transcript editing leads to cell migration and tumor invasion. However, Oakes et al. detected that ADAR3 expression was higher in glioblastoma compared to adjacent brain tissues. Meanwhile, they found that ADAR3 directly binds to GRIA2 precursor-mRNAs in astrocytes and astrocytoma cell lines. Overexpression of ADAR3 inhibits the RNA editing of the GRIA2 Q/R site, thus inhibiting tumor migration (Oakes et al., 2017). Interestingly, ADAR3 was also found to negatively correlate with the prognosis of low-grade gliomas, and positively correlate with GRIA2 (Q607R) editing. Zhang et al. believe that ADAR3 may inhibit the growth of glioma cells, and its high expression serves as a prognosis for patients with low-grade gliomas (Zhang et al., 2018). Therefore, the presence of ADAR3 may indicate a good prognosis for cancer patients.

## Adenine-Related RNA Modifications and Head and Neck Squamous Cell Carcinoma Therapy

The current treatment methods for HNSCC mainly include a combination of surgery, radiotherapy, chemotherapy, organ-sparing neoadjuvant radiotherapy and chemotherapy. There is an extreme treatment plan for recurrent or metastatic HNSCC (R/M HNSCC) cases that combines platinum, fluorouracil and cetuximab chemotherapy (Vermorken et al., 2008). This can increase the survival rate of patients (the risk of death ratio (HR) is 0.80, *p* = 0.04) (Leon et al., 2005; Stewart et al., 2009; Vermorken et al., 2014), but the efficacy of R/M HNSCC patients who are resistant to platinum-containing chemotherapy is limited. Also, many patients cannot tolerate the side effects caused by radiotherapy and chemotherapy. Therefore, it is necessary to find new immunotherapy targets.

HuR (ELAVL1) is an RNA-binding protein that plays a positive role in regulating tumor survival and invasion. Wang et al. proposed that knockout of HuR through CRISPR/Cas9 (HuR-CRISPR) inhibits tumor progression. Multifunctional nanoparticles can achieve targeted delivery of HuR CRISPR and epirubicin, and significantly improved the symptoms of mice bearing SAS tumors (Wang et al., 2021a). However, related research is still at the stage of animal experiments, and further research is needed to determine whether it can be applied to the clinic.

Programmed cell death protein-1 (PD-1/CD279) is an important immune checkpoint expressed by T cells. It can be coupled with the programmed death ligand PD-L1 (B7-H1/CD274) expressed by tumor cells and inhibit the activation of T cells and the anti-tumor response, evading immune system clearance. Immune checkpoint inhibitors (ICIs) can block the inhibitory immune checkpoint pathway, thereby reactivating anti-tumor immune activity. Common ICIs include PD-1/PD-L1 inhibitors that can specifically bind to PD-L1 on tumor cells and block the inhibitory immune checkpoint pathway (Gong et al., 2018). TIGIT is a new immune checkpoint molecule that binds to CD155 with high affinity (Yu et al., 2009). It can deliver immunosuppressive signals by binding to CD155 in competition with CD226 (dNaM-1). Liang et al. showed that CD155 (+) PD-L1 (+) bone marrow mesenchymal stem cells are enriched in the tumor microenvironment. Blocking TIGIT/CD155 combined with PD-L1 monoclonal antibody treatment can significantly improve the efficacy of HNSCC (Mao et al., 2021). Meanwhile, *Liu et al*. proposed that the expression of PD-L1 in HNSCC cytology samples is highly consistent with matched histological samples (Liu et al., 2021). Lee et al. also believe that ICIs show good anti-tumor activity against R/M HNSCC (Lee et al., 2021). Therefore, ICIs can be used for the treatment of HNSCC, and PD-1/PD-L1 inhibitors may play a positive role in promoting this treatment.

In addition, there are other immunotherapy approaches for HNSCC, such as p53 targeted therapy. Adenoviral p53 can work in conjunction with immune checkpoint inhibitors to jointly exert anti-cancer effects (Sobol et al., 2021). Type I interferon treatment can increase the amount and degree of RNA editing in ESCC cell lines (Zhang et al., 2017). Epigenetic therapy using the CRISPR-dCas9 method accurately targets and reactivates zygote arrest 1 (ZAR1), allowing it to regain its role as a tumor suppressor (Deutschmeyer and Richter, 2020). Patients with advanced HNSCC can receive anti-OX40 neoadjuvant treatment before surgery. It not only is safe, but can also increase the activation and proliferation of CD4^+^ and CD8^+^ T cells in the blood and tumors (Duhen et al., 2021) (Table 2). Therefore, we have reason to believe that the future treatment of HNSCC will be more accurate and effective.

**TABLE 2:** Immunotherapeutic methods and targets of HNSCCs.

Remedy	Regulation of target	Target	Mechanisms
CRISPR/Cas9 (HuR-CRISPR)	Knockout	HuR (ELAVL1)	The multifunctional nanoparticles Wang et al. designed can achieve targeted delivery of HuR CRISPR and epirubicin, and significantly improved the symptoms of mice bearing SAS tumors Wang et al. (2021a)
CRISPR-dCas9	Reactivate	ZAR1	Epigenetic therapy through the CRISPR-dCas9 method can accurately target and reactivate zygote arrest 1 (ZAR1), allowing it to regain its role as a tumor suppressor Deutschmeyer and Richter, (2020)
Immune Checkpoint Inhibitor (TIGIT)	Block	CD155	Blocking TIGIT/CD155 combined with PD-L1 monoclonal antibody treatment can significantly improve the efficacy of HNSCC Mao et al., 2021
Immune Checkpoint Inhibitor	Conjunct	P53	Adenoviral p53 can work in conjunction with immune checkpoint inhibitors to jointly exert anti-cancer effects Sobol et al., 2021
Immune Checkpoint Inhibitor	Inhibition	PD-1/ PD-L1	ICIs have good anti-tumor activity against R/M HNSCC Lee et al., 2021
Type I interferon	Promote	ADAR1	Type I interferon treatment can increase the amount and degree of RNA editing in esophageal squamous cell carcinoma cell lines Zhang et al., 2017
anti-OX40 neoadjuvant	Activate	CD4^+^ and CD8^+^ T cell	Patients with advanced HNSCC can receive anti-OX40 neoadjuvant treatment before surgery, which is not only safe, but can also increase the activation and proliferation of CD4^+^ and CD8^+^ T cells in the blood and tumors Duhen et al., 2021

## Discussion

RNA editing refers to post-transcriptional changes in RNA sequences. RNA modifications play key roles in the occurrence of various cancers, but the specific mechanisms are still unclear. These modifications may act as tumor-promoting factors to promote tumor growth, while it can also be used as inhibitors to limit the occurrence of tumors.

Presently, RNA editing research in HNSCC can be summarized into the following four mechanisms: 1) The number of RNA editing changes. An increase in the number of RNA edits can be observed in some cancers, but it does not cause overexpression of oncogenes. The may be due to the presence of these editing sites in normal tissues as well (Takahashi et al., 2006; Chen et al., 2013). 2) So far, most reported RNA edits are located in introns and 3′-UTRs, and there is almost no RNA edits present in the coding region (Zhang et al., 2016; Liu et al., 2019). These transcriptional “noises,” once thought to have no biological functions, have been shown to play a key role in some biological activities of eukaryotes, such as chromatin modification, post-transcriptional processing and nuclear transport (Ponting and Belgard, 2010; Nagano and Fraser, 2011; Lee, 2012; Shi et al., 2013). Some studies related to APA events have shown that some oncogenes have changes in the length of 3′-UTRs through APA events, thereby avoiding gene suppression and promoting tumor occurrence. A study reveals that m^6^A demethylase ALKBH5 is directly regulated by DDX3 which leads to decreased m^6^A methylation in FOXM1 and NANOG nascent transcript that contribute to chemoresistance (Shriwas et al., 2020). This mode of RNA editing has been studied (Murthy and Manley, 1995; Sun et al., 2014; Missan et al., 2015; Sun et al., 2017; Li et al., 2020b). 3) Even though tumor samples show simultaneous increases or decreases in RNA editing levels, changes in RNA editing in specific sites related to tumorigenesis show the opposite trend (Han et al., 2015; Paz-Yaacov et al., 2015). For example, breast cancer shows a significant increase in RNA editing at the transcriptome level, but in the specific editing sites affecting breast cancer, the RNA editing drops below 10% (Fumagalli et al., 2015). IGFBP7 transcripts are highly edited in the normal epidermis, while this editing is significantly reduced in basal cell and squamous cell carcinomas (Hochberg et al., 2013); 4) A-to-I is regulated by the ADAR enzyme RNA editing, thereby regulating the editing level of oncogenes and regulating the development of cancer (Qiao et al., 2014; Chen et al., 2017c; Chen et al., 2017d; Dong et al., 2018; Caponio et al., 2019; Yu et al., 2019). For example, ADAR1 can mediate the increase of A-to-I editing, resulting in over-editing of AZIN1 and promoting the development of ESCC (Qin et al., 2014).

In summary, recent studies have revealed RNA editing events in HNSCC. However, there is still very little known about the specific mechanisms of RNA modification to control cancer progression and drug resistance. Currently, investigators have been able to study the editing of non-coding regions using high throughput sequencing technology. At the same time, they have also found that non-coding regions have the highest levels of complexity. As a result, we started to explore the expression regulation of miRNAs and lncRNAs from a new perspective. This new concept breaks traditional thinking, which believes that unedited sequences or biomarkers must be linked to downstream targets through certain mechanisms. The mechanism by which RNA editing regulates the occurrence and development of cancer is still unclear. Research on the pathophysiological functions of RNA modifications in cancer is still in the early stages of research and there is still a long way to go. However, there is increasing evidence that the dysregulation of RNA editing central mediators (such as ADAR) contributes to the progression of cancer. Therefore, future work should focus on how to translate these modifications into available treatment options and how to relate these modifying behaviors to diagnosis and prognosis.

## Glossary

3' -UTR: 3′-untranslation regions
5′-UTR: 5′-untranslated region
ADARs: Adenosine deaminase acting on RNA
A-to-I editing: Adenosine-to-Inosine Editing
ALKBH5: AlkB homolog 5
ALKBH3: AlkB homolog 3
ASEs: Alternative splicing events
APA: Alternative Polyadenylation
R: Arginine
EGR1: Arly growth response 1
Asn: Asparagine
Asp: Aspartate
COAD: Colorectal Adenocarcinoma
CNV: Copy number variations
DDX3: DEAD-box RNA helicase 3
dsRNA: Double stranded RNA
DUSP1: Dual specificity phosphatase 1
ESCA: Esophageal carcinoma
ESCC: Esophageal squamous cell carcinoma
FTO: Fat mass and obesity-associated protein
FOXD1-AS1: Forkhead box D1 antisense RNA 1
GRIA2: Glutamate receptor ionotropic AMPA 2
HNSCCs: Head and neck squamous cell carcinoma
R/M HNSCC: Head and neck squamous cell carcinoma
HCC: Hepatocellular carcinoma
HPSCC: Hypopharyngeal squamous cell carcinoma
ICIs: Immune checkpoint inhibitors
IGFBP7: Insulin-like growth factor-binding protein-7
LSCC: Laryngeal squamous cell carcinoma
LIHC: Liver Hepatocellular carcinoma
mRNA: Messenger RNA
METTL3: Methyltransferase-like 3
METTL14: Methyltransferase-like 14
m^1^A: N1-Methyladenosine
m^6^A: N6-Methyladenosine
NPC: Nasopharyngeal carcinoma
ncRNAs: Non-coding RNAs
NSCLC: Non-small cell lung cancer
OSCC: Oral squamous cell carcinoma
PAAD: Pancreatic Adenocarcinoma
PTC: Papillary thyroid cancer
PD-1/CD279: Programmed cell death protein-1
RBM15: RNA-binding motif protein 15
RBM15B: RNA-binding motif protein 15B
STAD: Stomach Adenocarcinoma
TNBC: Triple-negative breast cancer
ZAR1: Zygote arrest 1
